# Supplementary material for: Olefin-Metathesis-Derived Norbornene–Ethylene–Vinyl Acetate/Vinyl Alcohol Multiblock Copolymers: Impact of the Copolymer Structure on the Gas Permeation Properties
Source: Polymers (Basel). 2022 Jan 22;14(3):444. doi: 10.3390/polym14030444 (PMC8839206; doi:10.3390/polym14030444)
Supplement: Supplementary file 1 [file polymers-14-00444-s001.zip › polymers-1549088-supplementary.pdf]

Supplementary Materials

Olefin-Metathesis-Derived Norbornene–Ethylene–Vinyl Acetate/Vinyl Alcohol Multiblock Copolymers: Impact of the Copolymer Structure on the Gas Permeation Properties

Alexey V. Roenko, Roman Y. Nikiforov, Maria L. Gringolts *, Nikolay A. Belov, Yulia I. Denisova, Georgiy A. Shandryuk, Galina N. Bondarenko, Yaroslav V. Kudryavtsev * and Eugene S. Finkelshtein

A.V. Topchiev Institute of Petrochemical Synthesis, Russian Academy of Sciences, Leninskii pr. 29, 119991 Moscow, Russia; royenko@ips.ac.ru (A.V.R.); nru@ips.ac.ru (R.Y.N.); belov@ips.ac.ru (N.A.B.); denisova@ips.ac.ru (Y.I.D.); gosha@ips.ac.ru (G.A.S.); bond@ips.ac.ru (G.N.B.); fin314@gmail.com (E.S.F.)
* Correspondence: gringolts@ips.ac.ru (M.L.G.); yar@ips.ac.ru (Y.V.K.)

The average lengths of N and COAc blocks in the multiblock copolymer, L_N and L_{COAc}, were calculated from the integral intensities of homo- (N–N, COAc–COAc) and hetero- (N-COAc, COAc–N) dyad signals in ^{13}C NMR spectra:

$$L_N = \frac{I\,(C = C_{N-COAc}) + I(C = C_{N-N})}{I(C = C_{N-COAc})};\ L_{COAc} = \frac{I\,(C = C_{COAc-N}) + I(C = C_{COAc-COAc})}{I(C = C_{COAc-N})}$$

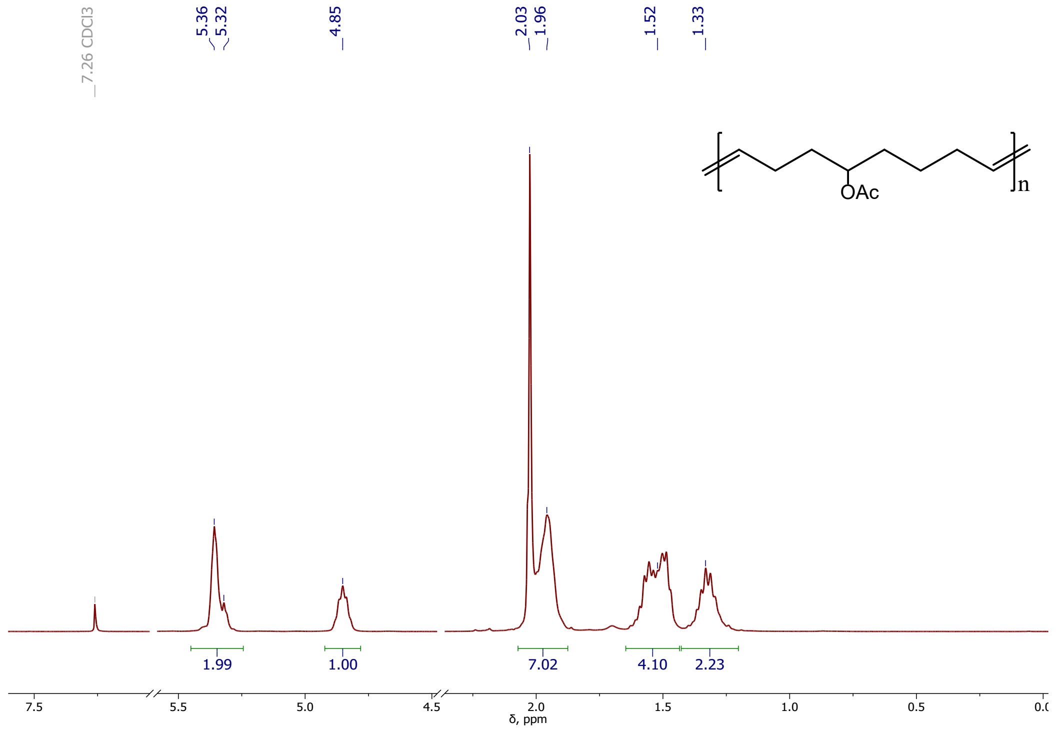

Figure S1. ^{1}H NMR spectrum of PCOAc at room temperature

^1H NMR (400.1 MHz, $CDCl_3$) δ, ppm: 5.36, 5.32 (2H, CH=CH); 4.86 (1H, HC-O-), 2.03– 1.96 (7H, CH3, CH2); 1.52 (4H, CH2); 1.34 (2H, CH2)

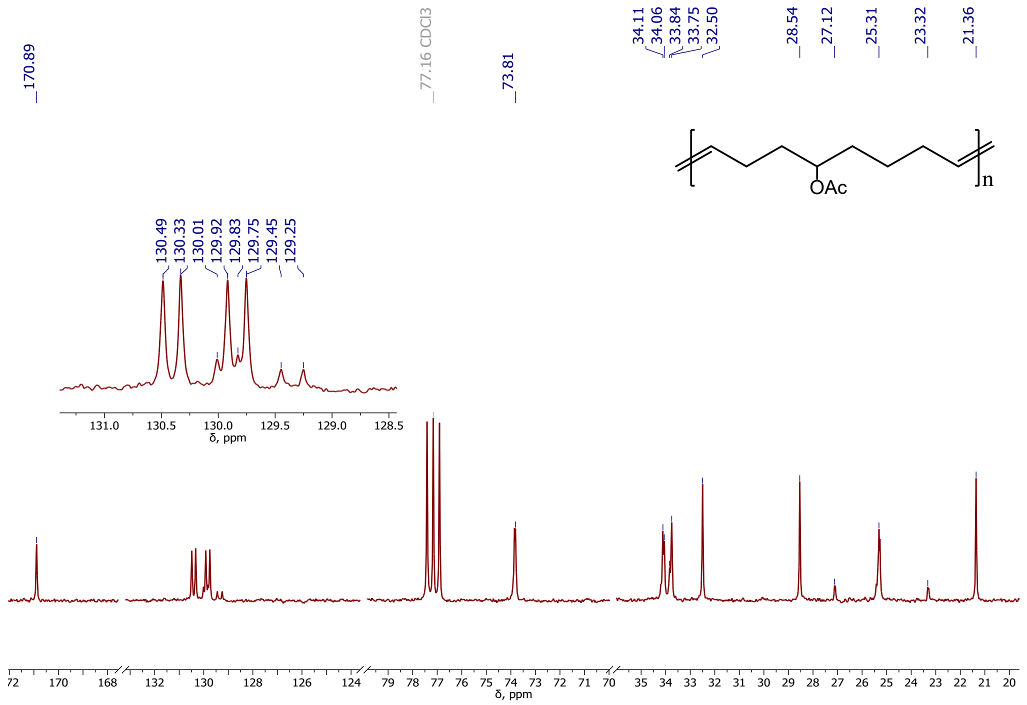

Figure S2. ^{13}C NMR spectrum of PCOAc at room temperature

^{13}C NMR (100.6 MHz, $CDCl_3$) δ, ppm: 170.89 (C=O); 130.52, 130.36, 129.95, 129.78 (trans CH=CH), 130.04, 129.86, 129.48, 129.28 (cis CH=CH); 73.94, 73.89, 73.85, 73.82 (HC-O-); 34.24, 34.17, 34.11, 33.97, 33.93, 33.89, 33.79, 32.53, 28.56, 27.16, 27.12, 25.48, 25.45, 25.42, 25.39, 25.35, 25.31, 23.36, 23.31 (CH_2); 21.37 (CH_3).

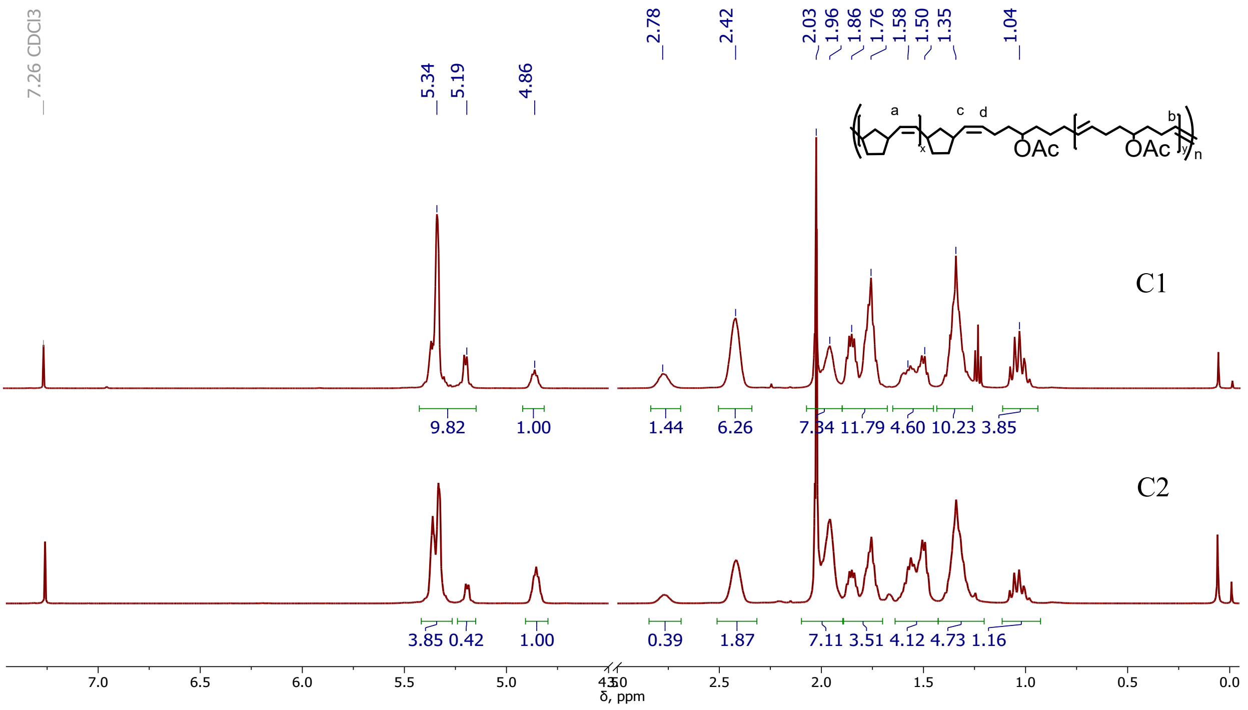

Figure S3. ^{1}H NMR spectra of (N-COAc)C at room temperature

^{1}H NMR (400.1 MHz, $CDCl_3$) δ, ppm: 5.36, (C^bH=CH, homodyads trans-COAc-COAc); 5.33 (C^aH=CH, homodyads trans-N-N; C^bH=CH, homodyads cis-COAc-COAc), 5.20 (C^aH=CH, homodyads cis-N-N), 4.86 (HC-O-, PCOAc), 2.77 (C***H***-CH=CH, cis-PN), 2.42 (C***H***-CH=CH, trans-PN), 2.03 (CH_3COO-, PCOAc), 1.96 (CH_2, PCOAc), 1.86, 1.76 (CH_2, PN), 1.58, 1.50 (CH_2, PCOAc), 1.35 (CH_2, PCOAc, PN), 1.04 (CH_2, PN).

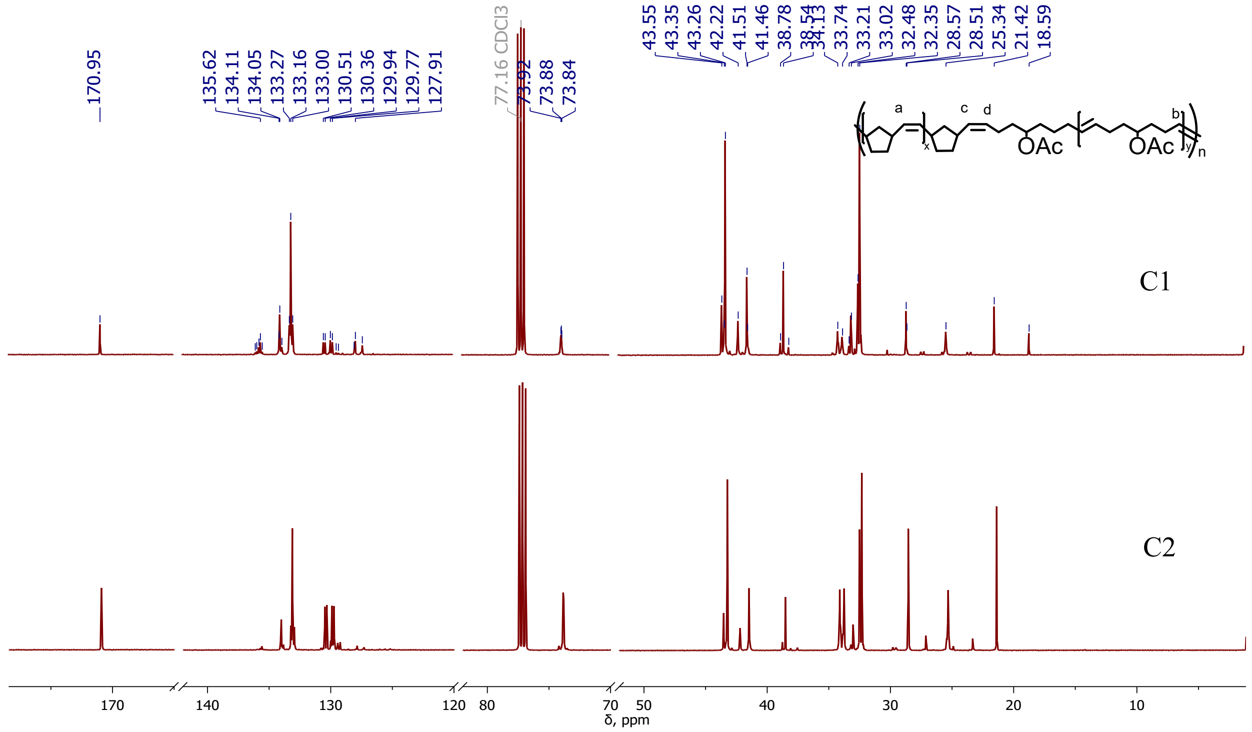

Figure S4. ^{13}C NMR spectra of (N-COAc)C at room temperature

^{13}C NMR (100.6 MHz, $CDCl_3$) δ, ppm: 170.95 (CH_3-*C*=O, PCOAc), 136.01, 135.90, 135.76, 135.62, 135.52, 135.49, 135.39, 135.34 (C^b=C, heterodyads N-COAc), 134.11, 134.05, 133.92 (C^a=C, homodyads cis-N-N), 130.51, 130.36, 129.94, 129.77 (C^b=C, homodyads trans-COAc-COAc), 130.03, 129.85, 129.47, 129.28 (C^b=C, homodyads cis-COAc-COAc), 127.98, 127.91, 127.41, 127.34 (C^d=C, heterodyads COAc-N), 73.88 (HC-O-, PCOAc), 43.55, 43.35, 43.26 (***C***H-CH=CH, trans-PN), 41.51, 41.46 (PN), 38.76, 38.54 (***C***H-CH=CH, cis-PN), 34.13, 33.74 (PCOAc), 33.21, 33.02 (PN), 32.48 (PCOAc), 32.35 (PN), 28.57, 28.51, 25.34 (PCOAc), 21.42 (***C***H3-COO-, PCOAc).

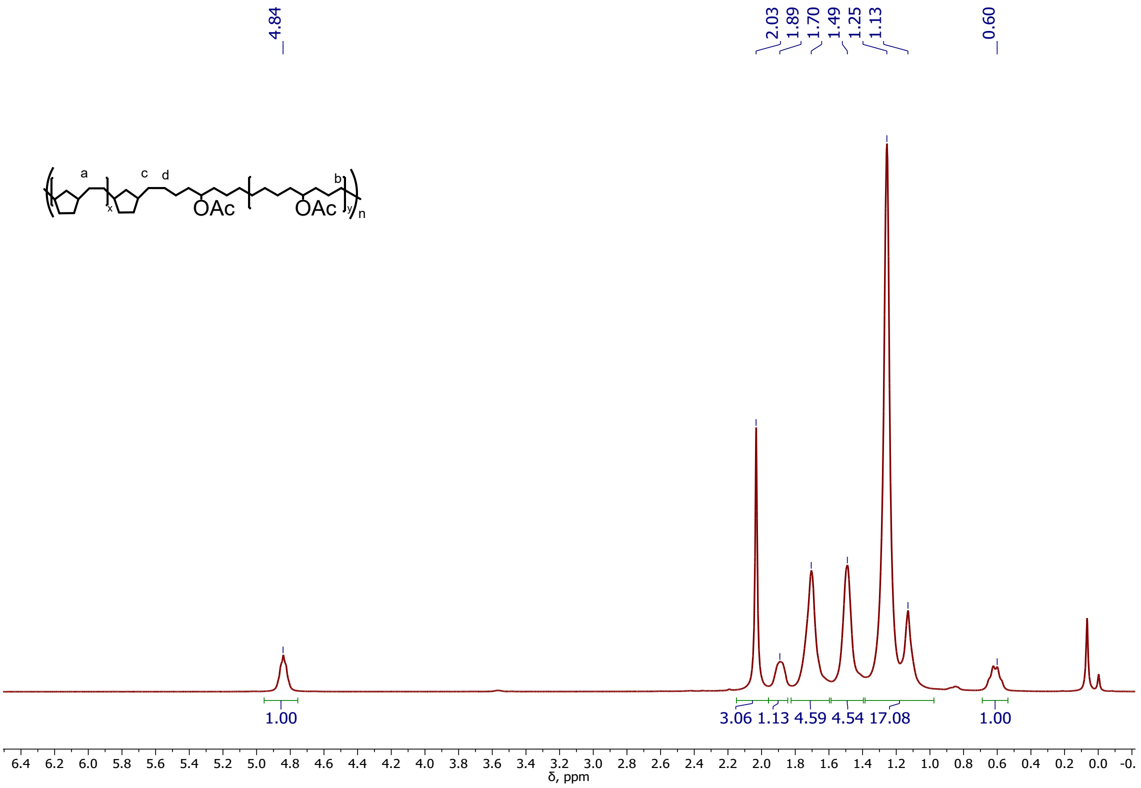

Figure S5. ^{1}H NMR spectra of H(N-COAc)C2 at room temperature

^{1}H NMR (400.1 MHz, $CDCl_3$) δ, ppm: 4.84 (HC-O-), 2.03 ($C\boldsymbol{H_3}COO$-), 1.89, 1.70, 1.49, 1.25, 1.12, 0.64-0.57 (CH_2).

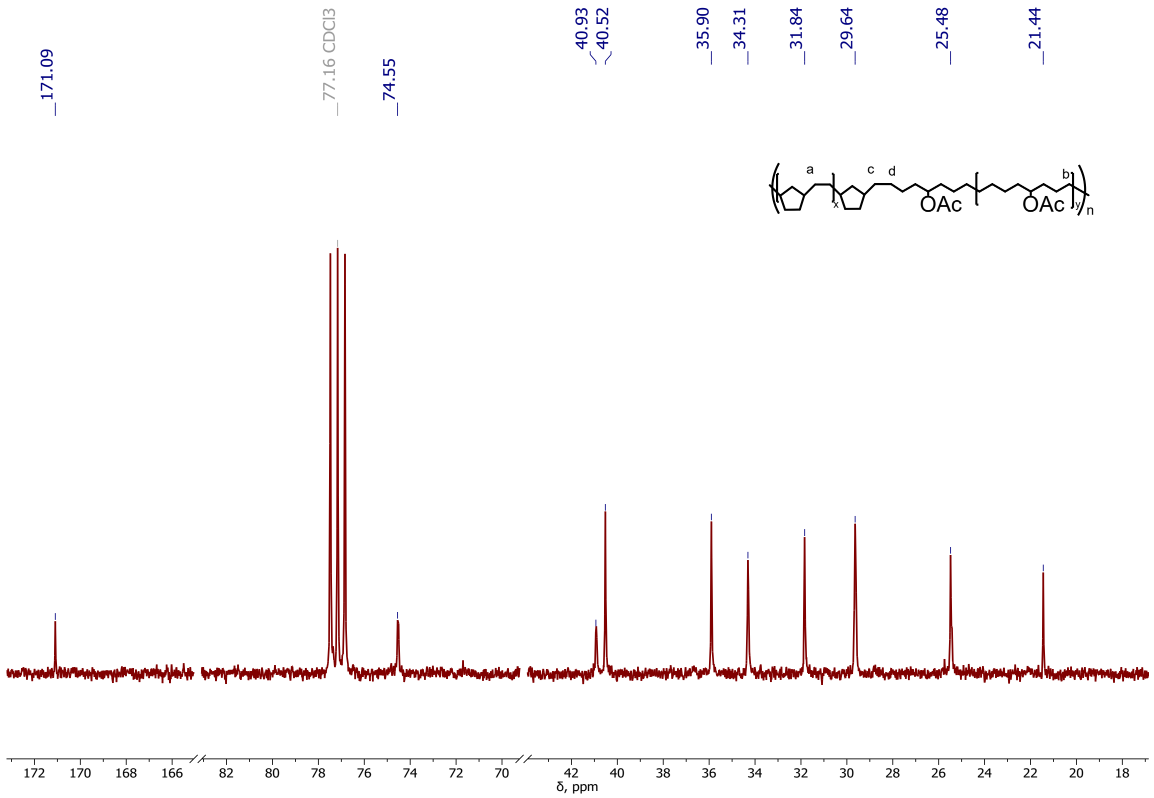

Figure S6.^{13}C NMR spectra of H(N-COAc)C2 at room temperature

^{13}C NMR (100.6 MHz, $CDCl_3$) δ, ppm: 171.09 (C=O), 74.55 (H***C***-O-), 40.93, 40.52, 40.24, 35.90, 34.31, 31.84, 29.64, 25.48, 21.44 (***C***H_2).

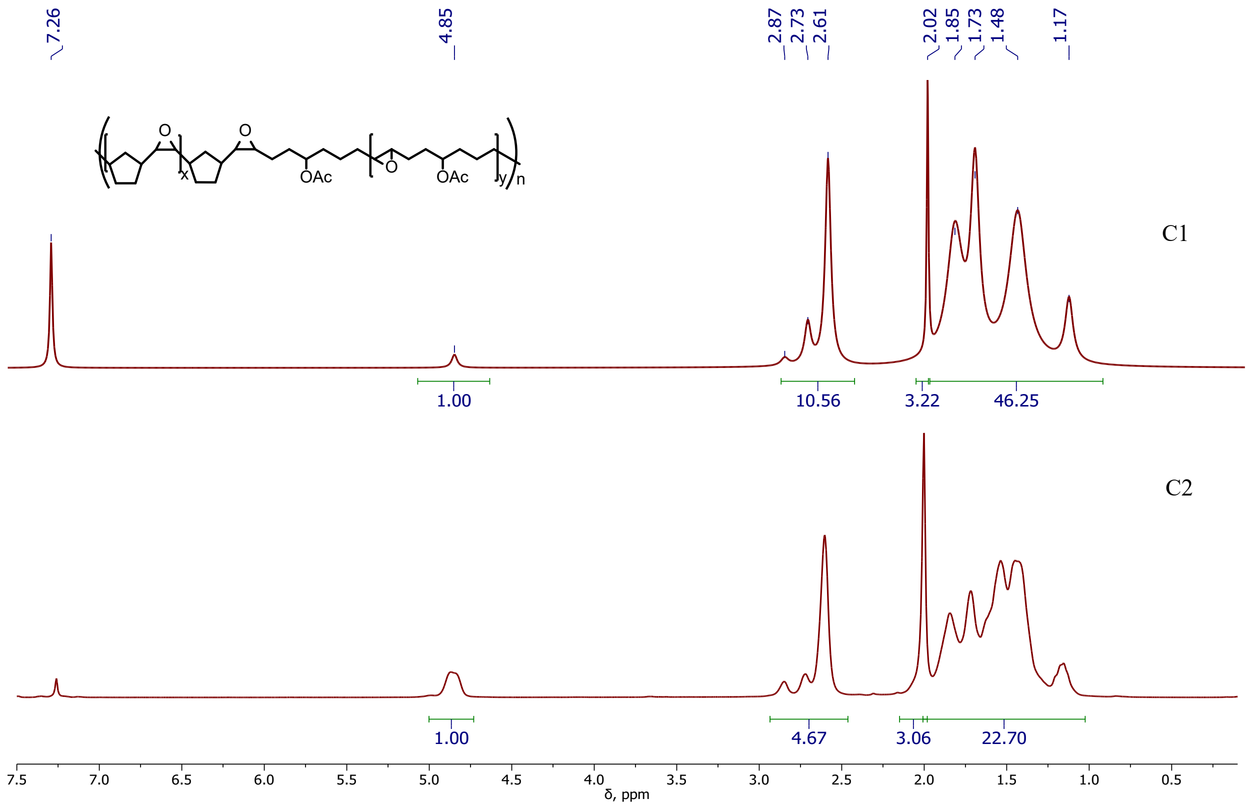

Figure S7. ^{1}H NMR spectra of E(N-COAc)C at room temperature

^{1}H NMR (400.1 MHz, $CDCl_3$) δ, ppm: 4.86 (***H***C-O-), 2.9-2.5 (C***H*** epoxy ring), 2.02 (CH_3COO-), 1.85, 1.73, 1.48, 1.17 (CH_2).

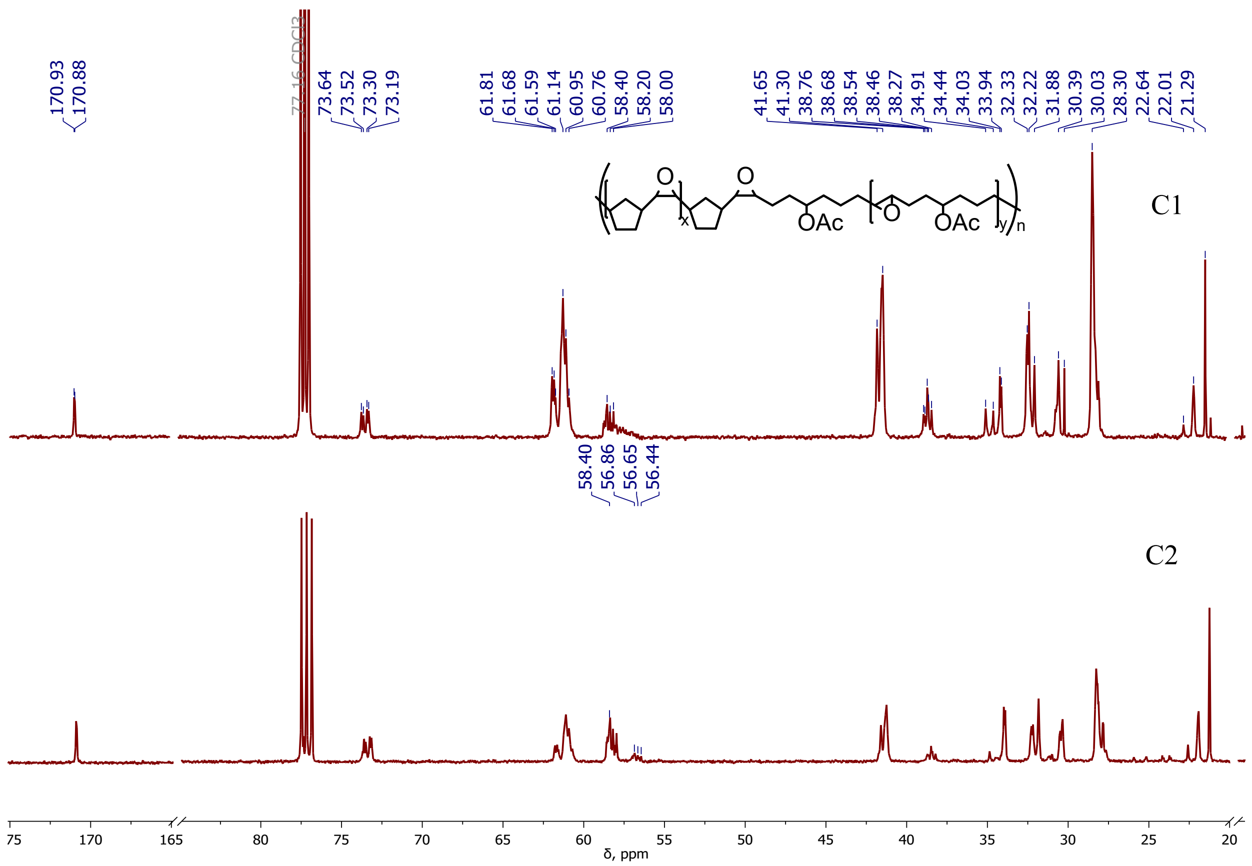

Figure S8. ^{13}C NMR spectra of E(N-COAc)C at room temperature

^{13}C NMR (100.6 MHz, $CDCl_3$) δ, ppm: 170.93, 170.88 (C=O), 73.64, 73.52, 73.30, 73.10 (H***C***-O-), 61.81, 61.68, 61.59 (cis-epoxy ring in N units), 61.14, 60.95, 60.76 (trans-epoxy ring in N units), 58.40, 58.20, 58.00 (trans-epoxy ring in COAc units), 56.86, 56.65, 56.44 (cis-epoxy ring in COAc units), 41.65, 41.30, 38.76, 38.68, 38.54, 38.46, 38.27, 34.91, 34.44, 34.03, 33.94, 32.33, 32.22, 31.88, 30.39, 28.30, 22.64, 22.01 (CH_2), 21.29 (***C***H_3C(O)O-).

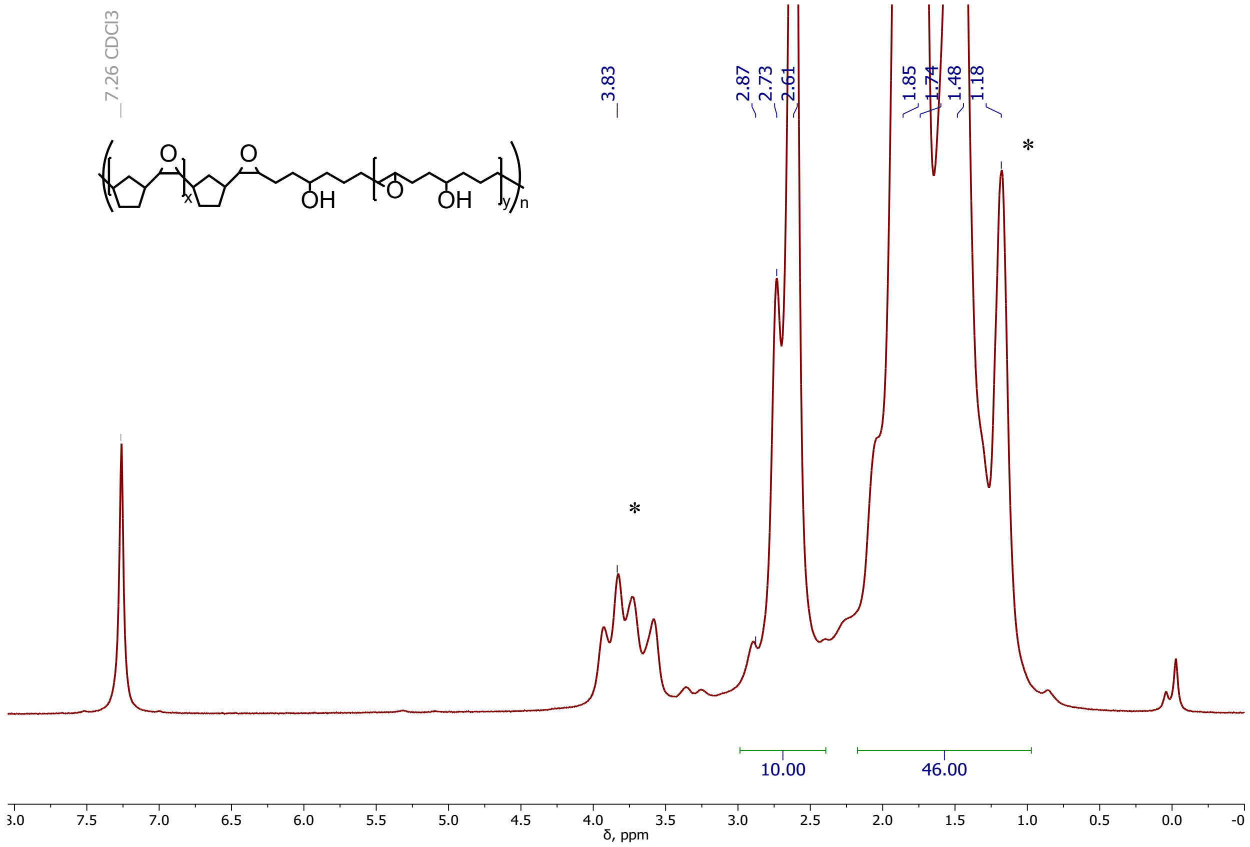

Figure S9. ^{1}H NMR spectra of E(N-COH)C1 at room temperature (*ethanol impurity)

^{1}H NMR (400.1 MHz, $CDCl_3$) δ, ppm: 3.83 (m, C***H***-OH), 2.87, 2.73, 2.61 (10H, m, C***H*** epoxy ring), 1.85, 1.74, 1.48, 1.18 (46H, m, CH_2).

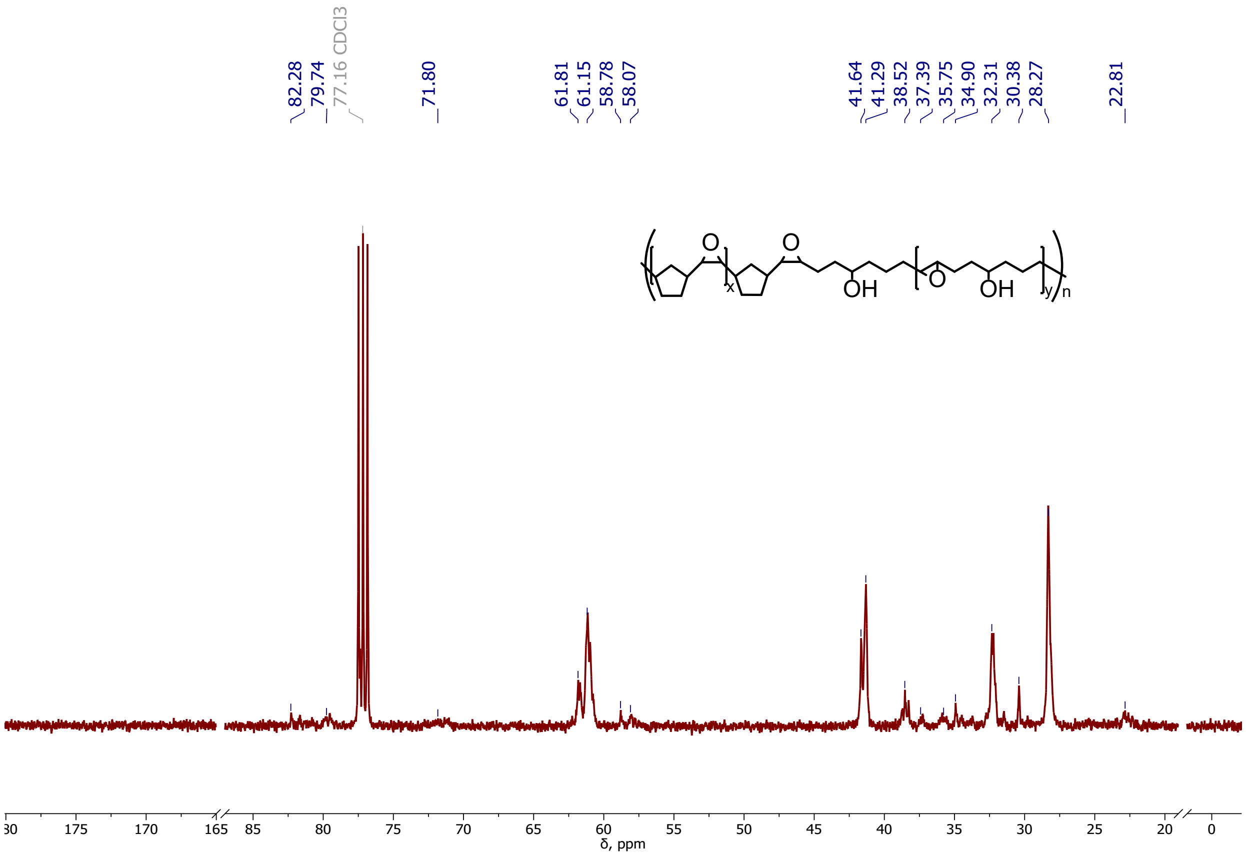

Figure S10. ^{13}C NMR spectra of E(N-COH)C1 at room temperature

^{13}C NMR (100.6 MHz, $CDCl_3$) δ, ppm: 82.28, 79.74 (C-O-C), 71.80 (C-OH), 61.81 (C-O-C of cis-epoxy ring in N units), 61.15 (C-O-C of trans-epoxy ring in N units), 58.78, 58.07 (C-O-C of epoxy ring in COH units), 41.64, 41.29, 38.52, 37.39, 35.75, 34.90, 32.31, 30.38, 28.27, 22.81 (CH_2).

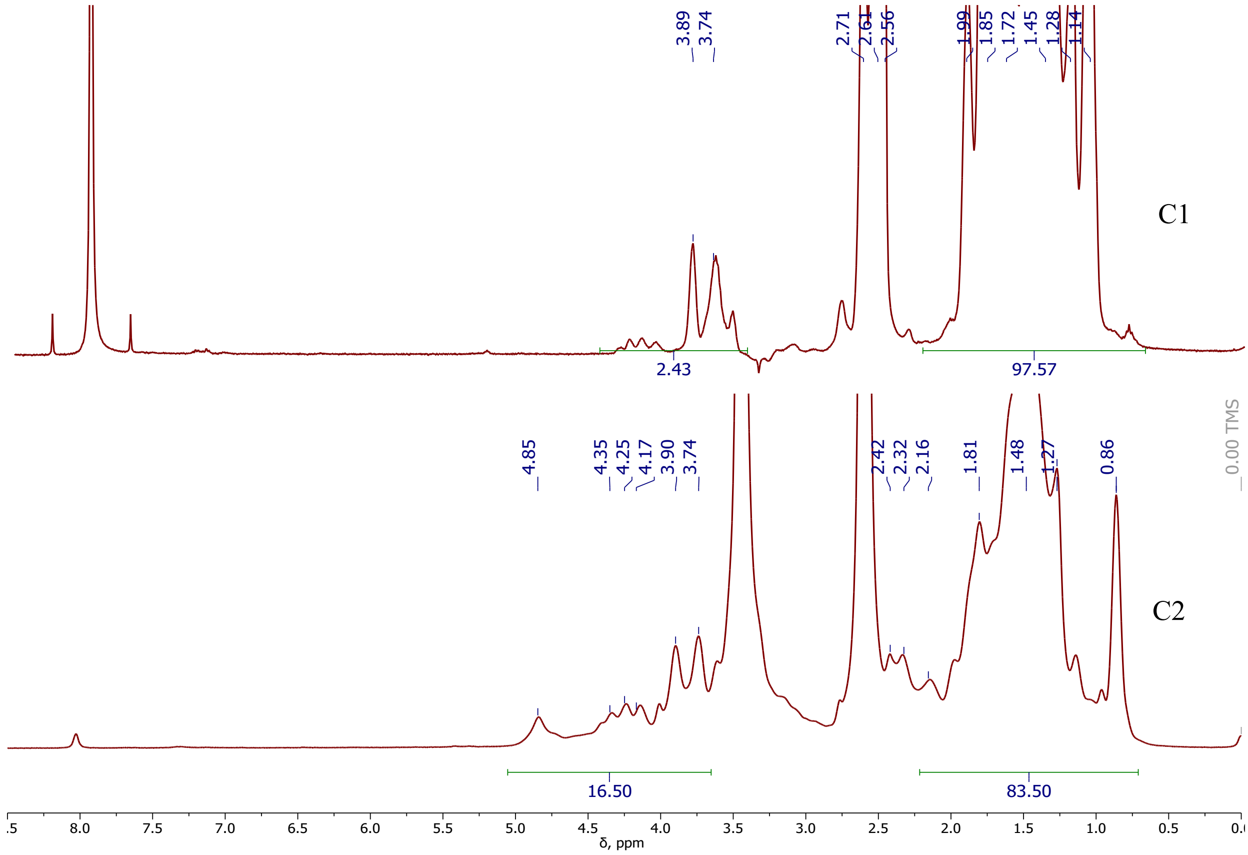

Figure S11. ^{1}H NMR spectra of E(N-COH)C at room temperature

^{1}H NMR (400.1 MHz, DMSO-d6:$CDCl_3$ 1:1 vol) δ, ppm (TMS): 3.89, 3.74, 2.71, 2.61, 2.56, 1.99, 1.85, 1.72, 1.45, 1.28, 1.14.

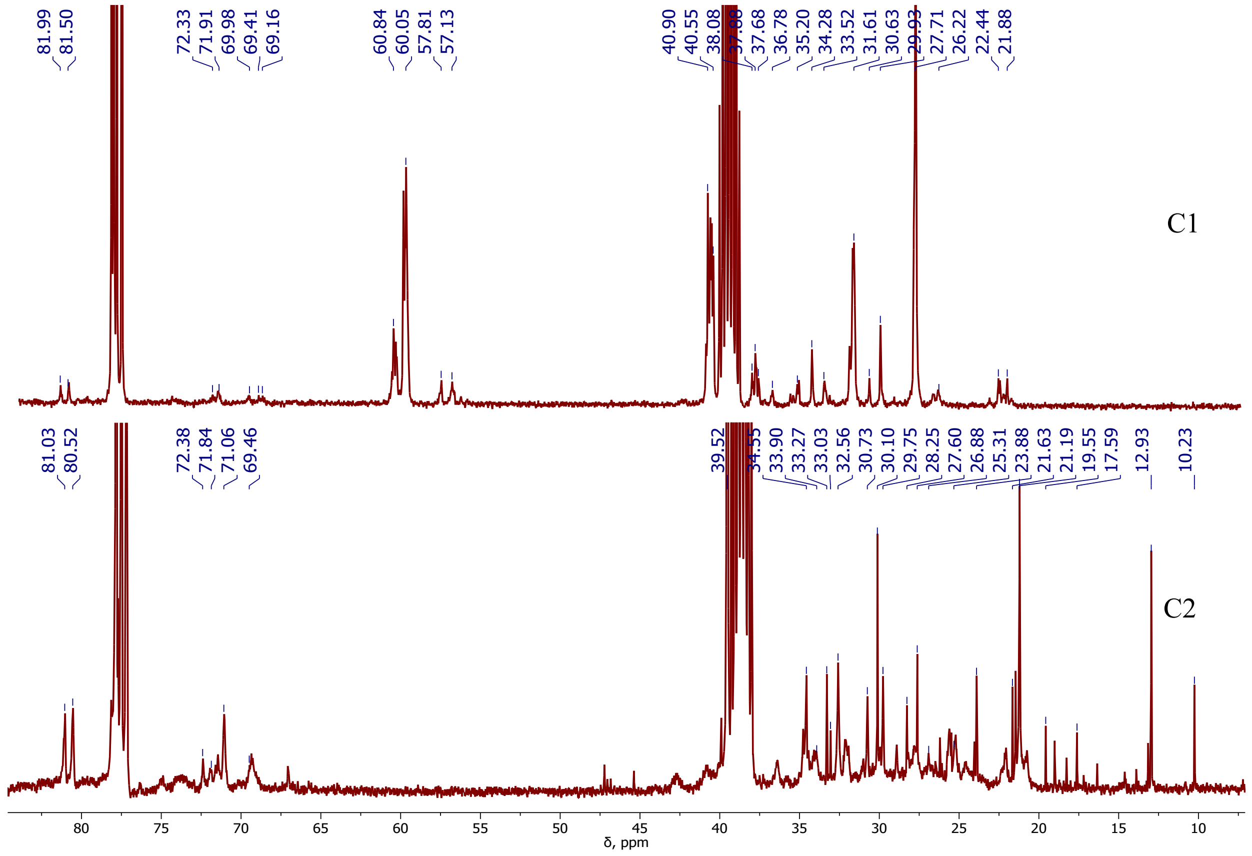

Figure S12.^{13}C NMR spectra of E(N-COH)C at room temperature

^{13}C NMR (100.6MHz, DMSO-d6:$CDCl_3$ 1:1 vol) δ, ppm (TMS): 81.99, 81.50 (C-O-C), 78.63, 72.33, 71.91, 69.98, 69.41, 69.16 (C-OH), 60.84 (C-O-C of cis-epoxy ring in N units) , 60.05 C-O-C of trans-epoxy ring in N units), 57.81 (C-O-C of trans-epoxy ring in COH units), 57.13(C-O-C of cis-epoxy ring in COH units), 40.90, 40.55, 38.08, 37.88, 37.68, 36.78, 35.20, 34.28, 33.52, 31.61, 30.63, 29.93, 27.71, 26.22, 22.44, 21.88.

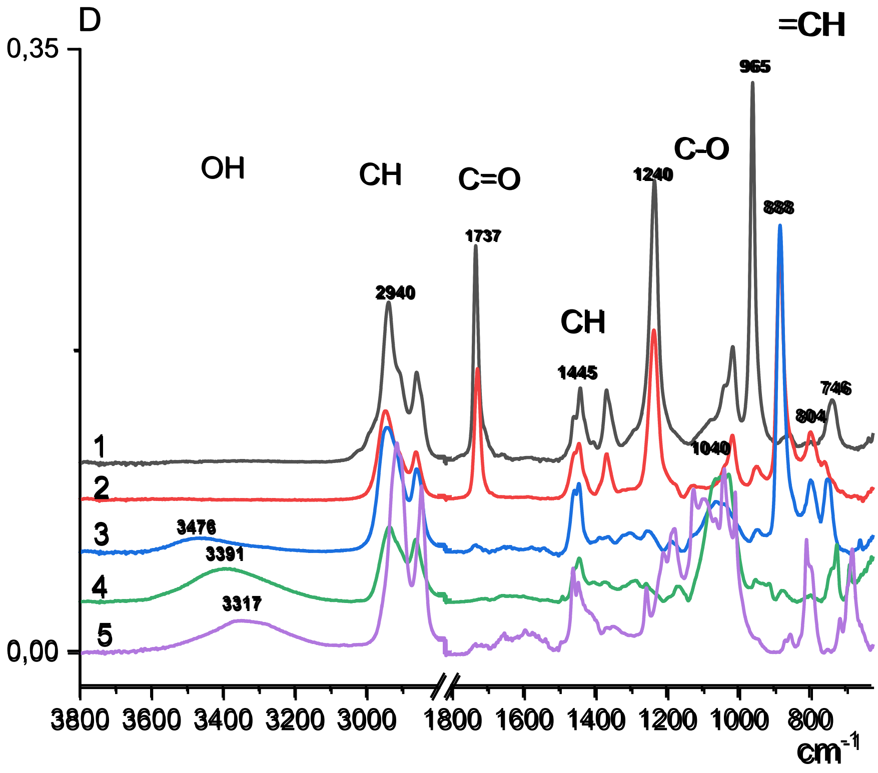

Figure S13. IR spectra of the synthesized copolymers: (1) (N-COAc)C1; (2) E(N-COAc)C1; (3) E(N-COH)C1; (4) E(N-COH)C2; (5) H(N-COH)C2

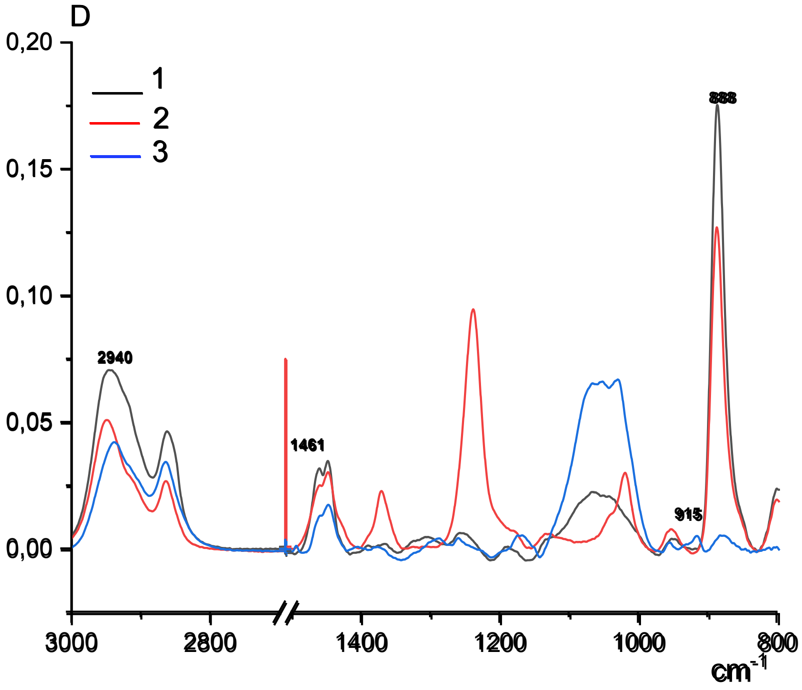

Figure S14. IR spectra of the synthesized copolymers:
(1) E(N-COAc)C1; (2) E(N-COH)C1; (3) E(N-COH)C2

Table S1. Relative intensities of the bands of epoxy groups (888 cm^{-1}) to the stretching (2940 cm^{-1}) and bending vibrations (1461 cm^{-1}) of -CH_2- groups

Copolymers	D888/D2940 · K*	D888/D1461 · K*
E(N-COAc)C1	0.562	1.171
E(N-COH)C1	0.557	1.169
E(N-COH)C2	0.091	0.192

*K – molar ratio [epoxy groups]/[CH_2] in copolymers: $K_{E(N\text{-}COAc)C1}$ = $K_{E(N\text{-}COH)C1}$ = 5/22= 0.227; $K_{E(N\text{-}COH)C2}$= 3/16=0.188

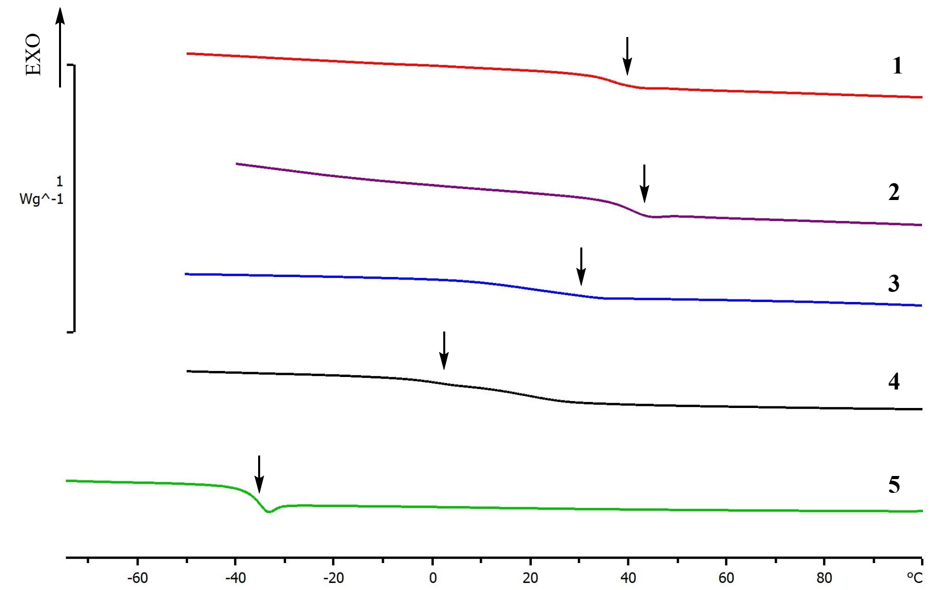

Figure S15. Second heating DSC curves of the synthesized polymers: (1) PN, (2) E(N-COH)C1, (3) E(N-COAc)C1, (4) – (N-COAc)C1, (5) – PCOAc. The arrows indicate the glass transition temperatures

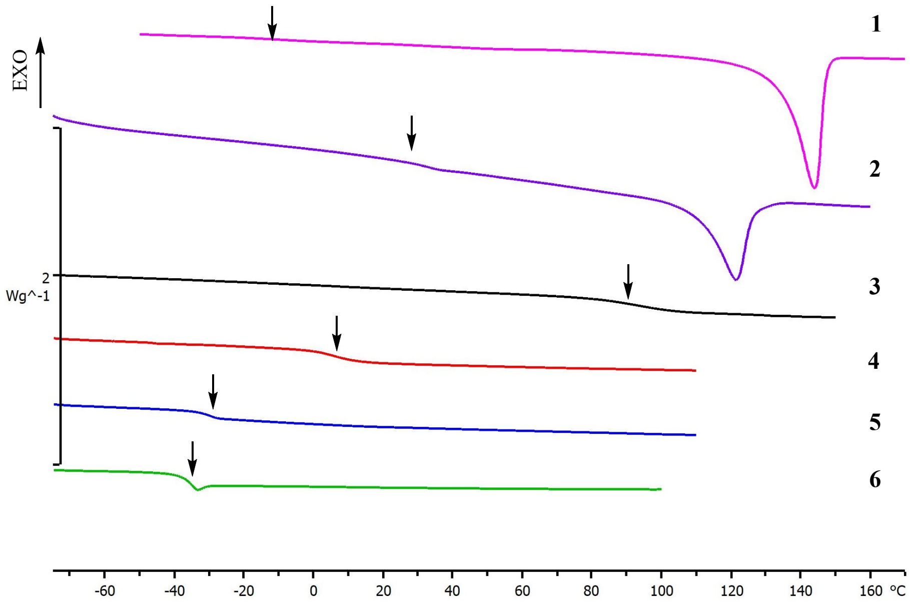

Figure S16. Second heating DSC curves of the synthesized polymers: (1) HPN, (2) H(N-COH)C2, (3) E(N-COH)C2, (4) E(N-COAc)C2, (5) (N-COAc)C2, (6) PCOAc. The arrows indicate the glass transition temperatures

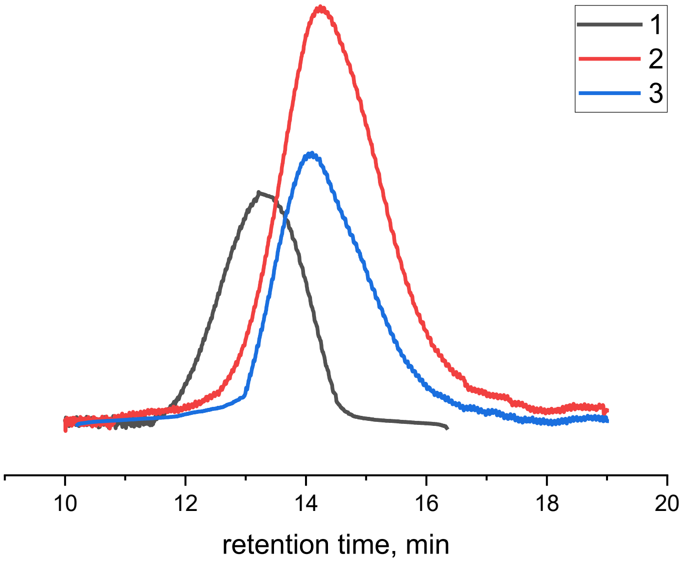

Figure S17. GPC curves of the starting and modified copolymers: 1 (N-COAc)C1, 2 E(N-COAc)C1, 3 E(N-COH)C1

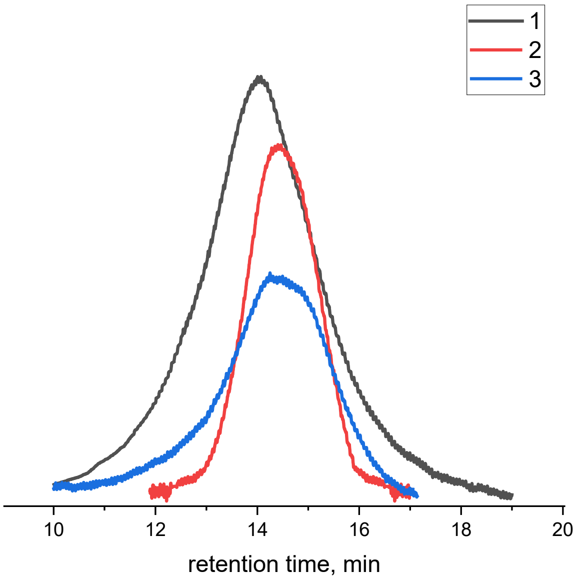

Figure S18. GPC curves of starting and modified copolymers: 1 – (N-COAc)C2; 2 – E(N-COAc)C2; 3 – E(N-COH)C2

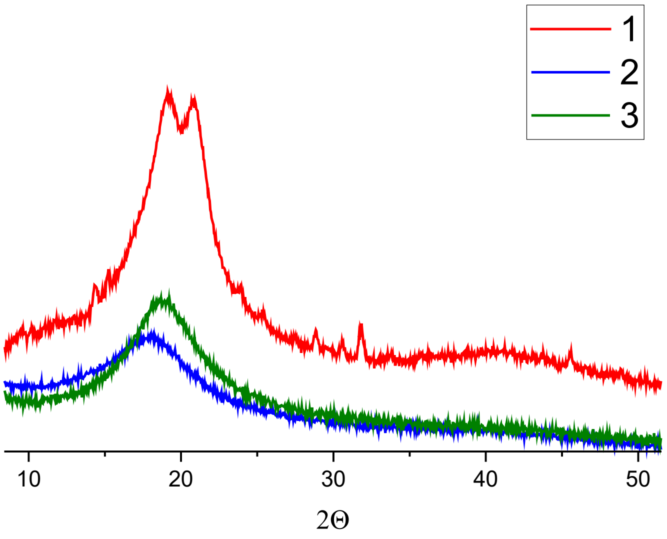

Figure S19. XRD diffraction patterns for some of the synthesized copolymers: 1 – H(N-COAc)C2; 2 – E(N-COH)C2; 3- E(N-COH)C1.
